# Integrated single-cell RNA-seq and bulk RNA-seq analysis to investigate key adipogenesis genes in adipose-derived stem cells

**DOI:** 10.1371/journal.pone.0335152

**Published:** 2025-12-01

**Authors:** Tongtong Zhang, Zhongming Cai, Haoran Li, Zhengyao Li, Leijuan Gan, Dali Mu

**Affiliations:** Department of Breast Plastic Surgery, Plastic Surgery Hospital, Chinese Academy of Medical Sciences and Peking Union Medical College, Beijing, China; Università degli Studi della Campania, ITALY

## Abstract

Adipogenic differentiation of adipose-derived stem cells (ADSCs) is fundamental to both adipose tissue homeostasis and clinical applications, particularly fat grafting. However, the global and stage-specific transcriptional regulatory networks underlying ADSC adipogenesis remain incompletely elucidated. In this study, we integrated bulk and single-cell RNA-seq datasets across multiple time points of ADSC adipogenesis to identify core regulators of differentiation and maturation. A total of 41 genes were consistently upregulated during early differentiation, among which eight hub genes (FABP4, FASN, FABP5, ADIPOQ, PLIN1, LPL, CIDEC, and ACSL1) formed a tightly connected protein–protein interaction (PPI) module associated with lipid metabolism, lipid droplet formation, and adipocyte maturation. Further integration of differentially expressed lncRNAs and miRNAs led to the construction of a ceRNA network involving 7 mRNAs, 9 miRNAs, and 4 lncRNAs, comprising 34 predicted lncRNA–miRNA–mRNA regulatory axes. To identify temporal transcriptional regulators, we defined five genes (TTC14, MBNL2, UBR3, ABCD2, and SORT1) as early-stage inducers of adipogenesis, and four genes (UQCR11, NDUFB4, S100A10, and PRDX3) as late-stage regulators involved in maintaining the mature phenotype. These stage-specific regulators showed distinct temporal expression patterns and were validated by qPCR. GeneMANIA network analysis further revealed that early-stage regulators were enriched in lipid transport and lipase activity regulation, while late-stage regulators were associated with mitochondrial electron transport and energy metabolism. These findings highlight the stage-dependent transcriptional landscape of ADSC adipogenesis and provide candidate regulatory targets for modulating adipocyte differentiation and stability.

## Introduction

Adipose-derived stem cells (ADSCs) have emerged as pivotal resources in regenerative medicine due to their multipotent differentiation capacity, immunomodulatory properties, and robust expansion potential, demonstrating indispensable value in tissue engineering and plastic reconstruction [1–4]. Significant clinical advancements have been achieved with ADSCs in fat grafting—a now established technique in cosmetic and reconstructive surgery for addressing volume and contour defects [5]. However, the primary clinical challenge associated with fat grafting is the viability and survival of the transplanted fat. Research indicates that promoting preadipocyte-to-adipocyte differentiation is pivotal for enhancing transplant viability, forming the basis of the “preadipocyte theory” [6,7]. Therefore, it is of vital importance to deeply understand the regulatory mechanism of adipogenic differentiation of ADSCs. In particular, there is an urgent need for a comprehensive analysis of the transcription factors and post-transcriptional regulators involved in adipogenesis to elucidate their specific roles and interactions within this process.

Multilayer regulatory networks precisely orchestrate the adipogenic differentiation of ADSCs. Core transcription factors PPARγ and C/EBPα serve as critical regulatory hubs indispensable for adipogenesis both in vitro and in vivo [8–12], while the novel regulator ATF4 positively modulates this process by enhancing C/EBPβ/Pol II binding at enhancer regions [13]. At the post-transcriptional level, miR-31 fine-tunes differentiation through negative regulation of the TINCR/C/EBPα feedback circuit [14], lncRNA H19 acts as a molecular sponge for miR-30a to relieve its suppression of target gene C8orf4 [15], and lncRNA-Adi activates the CDK6/pRb-E2F1 signaling cascade by sequestering miR-449a [16]. However, most of these studies have focused on the role of individual molecules, and the overall regulatory network involved in adipogenesis remains insufficiently explored. Unlike previous studies relying solely on bulk RNA-seq analysis, which cannot resolve cellular heterogeneity and transitional states, Integrating bulk and scRNA-seq at different differentiation time points enables a comprehensive analysis of the dynamic changes in gene expression during adipogenesis. thereby identifying stage-specific regulatory genes—those activated or maintained in a mature state during specific differentiation stages—remains an underexplored area, yet it is crucial for understanding the sequential logic of adipocyte lineage progression.

In this study, we performed an integrative analysis of bulk and single-cell RNA-seq datasets from ADSCs across multiple stages of differentiation. Through differential expression profiling, functional enrichment analysis, protein–protein interaction (PPI) network modeling, and ceRNA network reconstruction, we identified 8 core adipogenesis-related genes and elucidated their transcriptional and post-transcriptional regulatory circuits. Moreover, we uncovered stage-specific regulators associated with adipocyte induction and maintenance, reflecting dynamic and multilayered control of adipogenic progression. Together, these findings provide a comprehensive framework for understanding the regulatory architecture of adipogenesis and offer potential molecular targets for improving adipocyte differentiation and functional stability in fat grafting applications.

## Materials and methods

### Data collection

In this study, mRNA sequencing (mRNA-seq), microRNA sequencing (miRNA-seq), long non-coding RNA sequencing (lncRNA-seq), and single-cell RNA sequencing (scRNA-seq) were extracted from the Gene Expression Omnibus (GEO) database (https://www.ncbi.nlm.nih.gov/), which is maintained by the National Center for Biotechnology Information (NCBI). The mRNA data and LncRNA data were obtained from GSE61302 (https://www.ncbi.nlm.nih.gov/geo/query/acc.cgi?acc=GSE61302), which contains five undifferentiated ADSCs samples, four samples of adipocytes differentiated for seven days (early stage), and six samples of adipocytes differentiated for 21 days (late stage) [17]. The miRNA-seq data set, GSE25715 (https://www.ncbi.nlm.nih.gov/geo/query/acc.cgi?acc=GSE25715), contains four samples of differentiated ADSCs samples and four samples of adipocytes that were differentiated for three days. The scRNA-seq, GSE53638 (https://www.ncbi.nlm.nih.gov/geo/query/acc.cgi?acc=GSE53638), includes the following data: the single-cell RNA-seq data of undifferentiated ADSCs (Day 0, n = 8), adipocytes differentiated for seven days (Day 7, n = 14); and the single-cell RNA-seq data of adipocytes differentiated for 14 days (Day 14, n = 11). The three datasets were derived from different sequencing platforms. Specifically, GSE61302 (mRNA/lncRNA) was generated using the Affymetrix Human Genome U133 Plus 2.0 Array and normalized with MAS 5.0 via Affymetrix Expression Console. GSE25715 (miRNA-seq) was produced using the AB SOLiD System 3.0 and processed through the AB Small RNA pipeline v5.0, with normalization to one million total miRNA counts per sample. GSE53638 (scRNA-seq) was sequenced using the Illumina HiSeq 2000 platform and processed using the Seurat pipeline with log-normalization. Each dataset was normalized according to the platform-specific standard procedures to ensure data reliability and cross-platform comparability.

### Differentially expressed analysis

Due to variations in differentiation time, differentially expressed genes (DEGs) were identified in the mRNA-seq in a stage-specific manner. This stage-specific identification was performed between the early stage (day 7) and the undifferentiated stage, as well as between the last stage (day 21) and the early stage (day 7). respectively. In contrast to the focus on DEGs, this investigation specifically examined differentially expressed long non-coding RNAs (DE-lncRNAs) and differentially expressed microRNAs (DE-miRNAs) between the differentiated and undifferentiated stages. Differential expression analyses were conducted utilising the “limma” R package (version 3.46.0) with the wilcoxon test, which was implemented to identify differentially expressed genes with a |log2foldchange(FC) | > 0.5 and P-value < 0.05 [18]. The results were presented using volcano maps and heatmaps created with the “ggplot2” R package (version 3.4.1) and “ComplexHeatmap” R package (version 2.12.1), respectively.

### Single-cell analysis

Subsequent to the acquisition of the scRNA-seq data from the GEO database, a rigorous quality control (QC) procedure was implemented using the “Seurat” R package (version 4.0.5). This procedure entailed the removal of cells exhibiting a total number of genes exceeding 10, a gene expression level in fewer than 3 cells, and cells with a mitochondrial gene content of less than 10% [19]. The single-cell data processing followed established scRNA-seq normalization protocols using Seurat’s default LogNormalize method, which performs total count normalization with subsequent natural log transformation (counts per million scaled by 10,000). Following data normalisation, high-variance genes were extracted via FindVariableFeatures function, and the top 2,000 high-variance genes were selected via “vst” method. Principal component analysis (PCA) was then conducted for dimensionality reduction, and the top 30 components were included in further investigation. Subsequently, cluster analysis of the remaining cells was conducted depending on the time of differentiation, and the results were visualized via uniform manifold approximation and projection (UMAP). Finally, with an adjusted *P*-value < 0.05, the FindMarkers function with Wilcoxon test was employed to identify the DEGs in day 7 *vs.* day 0 and day 14 *vs.* day 7.

### Functional enrichment analysis

The “clusterprofiler” R package (version 4.4.4) and the org.Hs.e.g.,db (version 3.15.0) were utilized for Gene Ontology (GO), incorporating biological processes (BP), molecular functions (MF), and cellular components (CC), as well as Kyoto Encyclopedia of Genes and Genomes (KEGG). The results were then visualized using the “GOplot” R package (version 1.0.2) and tree map (version 2.4-3) [20,21].

### Construction of protein-protein interaction (PPI) network

In order to investigate the potential interaction of proteins, the STRING database (https://string-db.org) was utilized to establish protein-protein interaction (PPI) networks with a confidence threshold of 0.4. The results were then displayed via Cytoscape. Two distinct Cytoscape plug-ins were employed: (1) MCODE for network module detection (parameters: Degree Cutoff = 2, Node Score Cutoff = 0.2, Haircut = true, Fluff = false, K-Core = 2, Max. Depth = 100), and (2) CytoHubba for topological analysis to identify hub genes. Finally, we combined the results of MCODE and CytoHubba were combined to identify the hub genes.

### Establishment of a ceRNA network

The prediction of regulated hub genes was conducted using multiMiR (version 1.18.0), with the identification of interacting long non-coding RNAs (lncRNAs) with the predicted microRNAs subsequently facilitated by the Starbase database (https://starbase.sysu.edu.cn/index.php) (parameters: clipExpNum ≥ 2, pancancerNum ≥ 1, and geneType = lncRNA). Subsequently, based on the principle that miRNAs exhibit opposite expression patterns to mRNA expression, and lncRNAs is in line with the trend of mRNA expression, the common miRNAs or lncRNAs were obtained by intersecting differentially expressed (DE)-miRNAs with predicted miRNAs, and differentially expressed (DE)-lncRNAs with predicted lncRNAs, respectively. The final step in the process was to create and visualise a ceRNA network using Cytoscape.

### Identification of ADSCs

The trilineage differentiation potential of adipose-derived mesenchymal stem cells was assessed as previously described. All the chemicals were purchased from Oricell. Osteogenic differentiation was induced by incubating adipose-derived mesenchymal stem cells in culture media supplemented with 10 mM β-glycerophosphate, 50 μM ascorbic acid, and 0.1 μM dexamethasone. The medium was replaced every three days for three weeks, and alizarin red staining was performed. Adipose-derived mesenchymal stem cells were induced in culture media supplemented with 0.5 mM 3-isobutyl-1-methylxanthine, 10 μM insulin, 200 μM indomethacin, and 1 μM dexamethasone. Three weeks after induction, cells were incubated for 30 min in 0.5% (weight/volume) oil red O. For chondrogenic differentiation, the cells were incubated in culture media supplemented with 1 mM sodium pyruvate, 1% insulin–transferrin sodium selenite, 0.17 mM ascorbic acid, 0.35 mM l-proline, 1.25 mg/ml bovine serum albumin, 5.33 μg/ml linoleic acid, 0.1 μM dexamethasone, and 0.01 μg/ml TGFβ. The medium was replaced every three days for four weeks, and the cells were assessed by toluidine blue staining.

### qPCR verification of expression levels

For RNA analysis, total RNA was extracted with TRIzol (Thermo Fisher Scientific), followed by complementary DNA synthesis with Hiscript III RT SuperMix for quantitative polymerase chain reaction (qPCR) (+gDNA wiper) (Vazyme, R323-01). Reverse transcription qPCR was conducted using the THUNDERBIRD SYBR qPCR Mix (TOYOBO, QPS-201) on a CFX384 Real-Time PCR system (Bio-Rad Laboratories). The relative mRNA expression level of each gene was normalized to GAPDH expression, calculated using the ∆∆Ct method.

## Results

### Dynamic transcriptome analysis identifies a core gene set driving adipogenic differentiation

**Fig 1:**
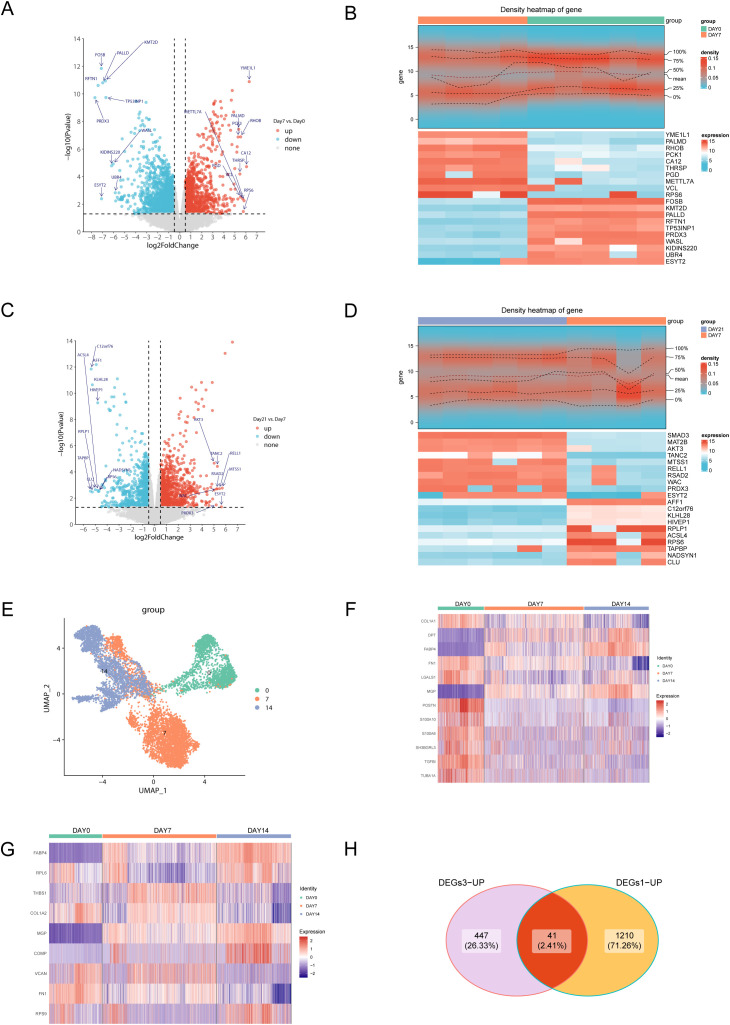
Identification of adipogenesis-related genes. **(A)** A volcano plot illustrating the DEGs between the early stage and the undifferentiated stage **(B)** A heatmap plot showing the top 10 up-regulated genes and down-regulated genes in the early stage **(C)** A volcano plot illustrating the DEGs between the last stage and the early stage **(D)** A heatmap plot showing top 10 up-regulated genes and down-regulated genes in the last stage **(E)** UMAP of cell clusters **(F)** A heatmap plot showing the expression of DEGs between day 7 and undifferentiated cells in day 0, day 7, and day 14 in GSE53638 **(G)** A heatmap plot showing the expression of DEGs between day 14 and day 7 in day 0, day 7, and day 14 in GSE53638 **(H)** Venn diagram showing adipogenesis-related genes.

To investigate the transcriptional regulatory mechanisms underlying adipogenic differentiation, we performed transcriptomic profiling of adipose-derived stem cells (ADSCs) at different stages of differentiation. Initially, by comparing bulk RNA-seq data from the undifferentiated stage (Day 0) and the early differentiation stage (Day 7), we identified a set of early adipogenesis-associated differentially expressed genes (DEGs1), comprising 1,251 significantly upregulated and 1,428 downregulated genes (Fig 1A). Representative upregulated genes included FOSB, KMT2D, RFTN1, and PRDX3 (Fig 1B). Among them, FOSB, a member of the AP-1 transcription factor family, has been reported to play a critical role in adipogenesis, while PRDX3 promotes lipid droplet formation and mitigates oxidative stress, supporting adipocyte differentiation [22,23]. Conversely, downregulated genes such as RHOB and THRSP are associated with the repression of preadipocyte activity and progression toward mature adipocytes, suggesting that metabolic reprogramming and cytoskeletal remodeling are already initiated at this stage.

Further transcriptomic comparison between the late differentiation stage (Day 21) and the early stage (Day 7) identified a second set of DEGs (DEGs2) related to terminal adipocyte maturation, consisting of 1,009 upregulated and 851 downregulated genes (Fig 1C). Among the upregulated genes, ACSL4 (Fig 1D) plays a pivotal role in lipid metabolism and fatty acid activation, contributing to the establishment of mature adipocyte function. Meanwhile, the downregulation of SMAD3 and AKT3 may reflect attenuation of the TGF-β and PI3K/AKT signaling pathways, potentially favoring the maintenance of terminal differentiation homeostasis.

To dissect the dynamics of adipogenic differentiation at single-cell resolution, we performed scRNA-seq on ADSCs collected at Days 0, 7, and 14. After stringent quality control (S1 Fig), three major cell clusters were identified. Undifferentiated ADSCs displayed distinct transcriptomic signatures, while a transitional state emerged between Day 7 and Day 14, indicating rapid differentiation progression. By Day 14, the clusters became more mature and transcriptionally distinct, suggesting the establishment of a stable adipocyte identity (Fig 1E).

Differential expression analysis across single-cell stages revealed 1,513 DEGs between Day 0 and Day 7 (DEGs3, Fig 1F), and 3,127 DEGs between Day 7 and Day 14 associated with late-stage differentiation (DEGs4, Fig 1G). To identify robust early adipogenic regulators, we intersected the upregulated genes from DEGs1 and DEGs3, yielding a set of 41 core genes consistently upregulated in early differentiation (Fig 1H). These genes are broadly involved in fatty acid metabolism, oxidative stress response, signal transduction, and lipid droplet formation, providing multi-dimensional evidence for the molecular underpinnings of adipogenesis.

### Core adipogenesis genes regulate lipid metabolism, redox balance, and thermogenesis

To further elucidate the functional characteristics of the 41 identified adipogenesis-related core genes, we performed Gene Ontology (GO) and Kyoto Encyclopedia of Genes and Genomes (KEGG) pathway enrichment analyses to uncover their potential biological roles in adipocyte formation and regulation.

**Fig 2:**
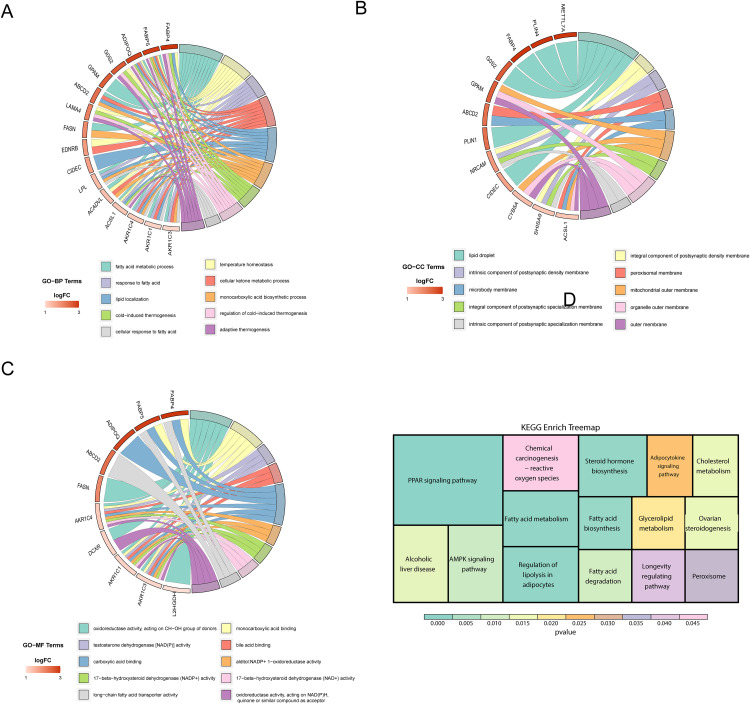
Functional enrichment analysis of adipogenesis-related genes. **(A)** Chordal graph of enriched GO BP terms **(B)** Chordal graph of enriched GO CC terms **(C)** Chordal graph of enriched GO MF terms **(D)** Treemap of enriched KEGG pathways.

In the GO biological process (GO-BP) analysis, these core genes were significantly enriched in multiple lipid metabolism-related pathways, including fatty acid metabolism, lipid localization, lipid droplet formation, and fatty acid stimulus response (Fig 2A, S1 Table). Notably, enrichment was also observed in cold-induced thermogenesis, temperature homeostasis regulation, and adaptive thermogenesis pathways associated with environmental stress responses, suggesting that these genes may participate not only in lipid metabolism but also in the adaptation of adipocytes to temperature fluctuations. For example, genes such as FABP4, FABP5, and ADIPOQ are widely involved in fatty acid binding and storage, while CIDEA, LPL, and ACADVL contribute to fatty acid oxidation and energy balance, constituting a complete pathway module from fatty acid uptake and transport to utilization.

GO cellular component (GO-CC) analysis indicated that these genes are predominantly localized to subcellular structures highly relevant to lipid metabolism, such as lipid droplets, peroxisomal membrane, mitochondrial outer membrane, and microbody membrane (Fig 2B). Among these, lipid droplets showed the most significant enrichment, including key regulatory factors such as PLIN1, FABP4, and CIDEC, emphasizing their central role in the regulation of lipid droplet formation and lipid storage. Additionally, significant enrichment was observed in synaptic post-synaptic density membranes and other neuronal structures, implying the possible involvement of neuronal signaling in adipogenesis, warranting further investigation.

GO molecular function (GO-MF) analysis highlighted the importance of these genes in redox regulation, fatty acid binding, and steroid metabolism (Fig 2C). Specifically, AKR1C1, AKR1C3, and AKR1C4 showed abundant oxidoreductase activity, implicating their roles in fatty acid oxidation and cholesterol metabolism. FABP4 and FABP5 were associated with monocarboxylic acid and bile acid binding, suggesting potential functions in fatty acid transport and cholesterol derivative regulation. Genes such as ABCD2 may mediate long-chain fatty acid transmembrane transport, supporting the material supply necessary for lipid accumulation.

KEGG pathway enrichment analysis identified 15 significantly enriched pathways (Fig 2D, S2 Table), encompassing multiple key steps in adipogenesis. Core pathways such as PPAR signaling, fatty acid metabolism, fatty acid synthesis and degradation, lipid droplet formation, and regulation of lipolysis in adipocytes were highly enriched, collectively outlining a molecular framework for adipocyte development. Among these, the “regulation of lipolysis in adipocytes” pathway was particularly notable, featuring key genes including FABP4, PLIN1, and CIDEC, which are involved in fatty acid uptake, maintenance of lipid droplet membrane integrity, and enhancement of lipid storage, respectively. These genes form a tripartite regulatory module for lipid metabolic homeostasis [24–27]. Furthermore, enrichment of the AMPK signaling pathway and adipocytokine signaling pathway underscores the role of these genes in energy sensing and the regulation of insulin sensitivity in adipose tissue. The involvement of genes related to cholesterol metabolism, steroid hormone biosynthesis, and oxidative stress responses further suggests that adipocyte differentiation may be modulated by a complex endocrine regulatory network, indicating potential systemic metabolic impacts.

The 41 core adipogenesis-related genes collectively contribute to a multidimensional regulatory network that supports adipocyte formation and functional establishment through the coordinated modulation of fatty acid metabolism, redox balance, thermogenesis, and endocrine processes.

### Interconnected protein and ceRNA networks identify Hub genes driving adipogenesis

**Fig 3:**
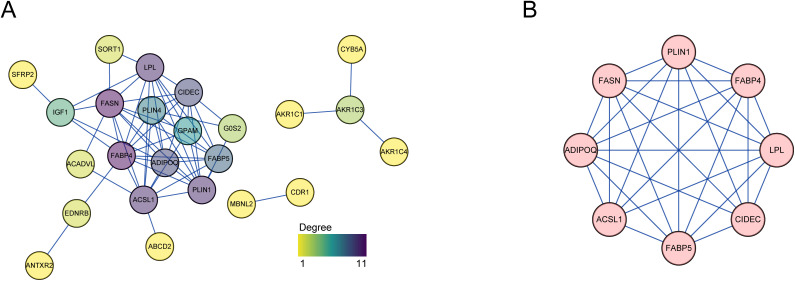
Identification of hub genes for adipogenesis. **(A)** The PPI network indicating the interactions of 24 proteins **(B)** The genes of the core gene module as the hub genes for further investigation.

To further dissect the functional interplay among adipogenesis-associated genes, we constructed a protein–protein interaction (PPI) network of the 41 core adipogenic genes using the STRING database, comprising 24 nodes and 58 high-confidence interaction edges (Fig 3A). To identify key functional modules within the network, we employed the MCODE (Molecular Complex Detection) algorithm, which delineated a highly interconnected core module consisting of eight pivotal genes (FABP4, FASN, FABP5, ADIPOQ, PLIN1, LPL, CIDEC, and ACSL1) (Fig 3B). These genes are extensively involved in fatty acid biosynthesis (FASN), lipid binding and storage (FABP4, FABP5, PLIN1), regulation of lipolysis (LPL, CIDEC), and metabolic homeostasis (ADIPOQ, ACSL1), forming a functionally integrated central network in the adipogenic process. Their high degree of interconnectivity supports their critical role in adipocyte maturation and the establishment of cellular functionality.

**Fig 4:**
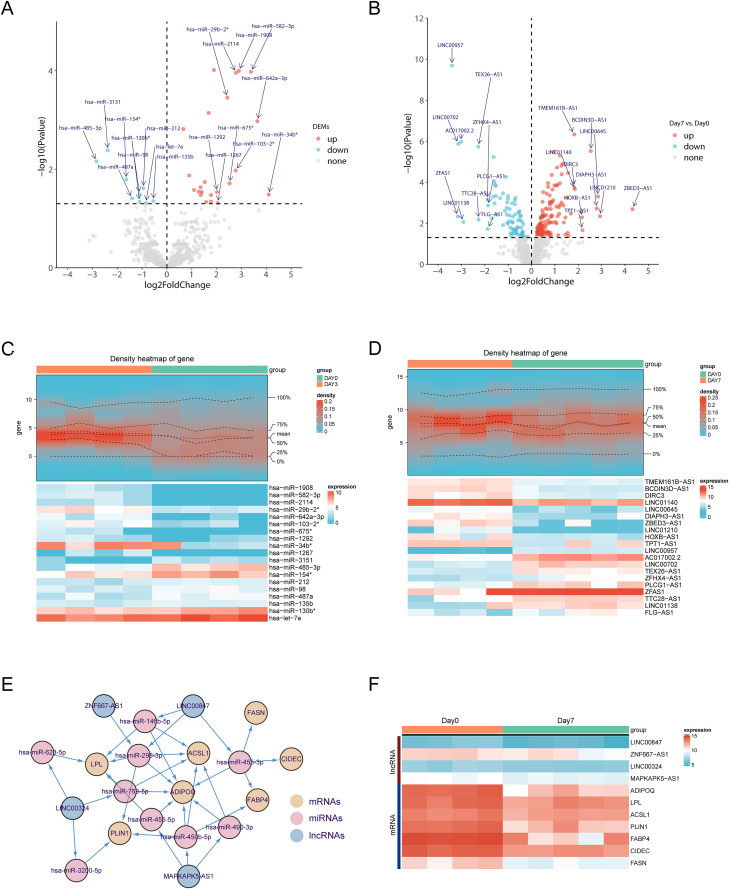
Construction of ceRNA network. **(A)** A volcano plot illustrating the DE-miRNAs between the differentiated stage and the undifferentiated stage **(B)** A volcano plot illustrating the DE-lncRNAs between the differentiated stage and the undifferentiated stage **(C)** A heatmap plot showing top 10 up-regulated and down-regulated miRNAs in the differentiated stage **(D)** A heatmap plot showing top 10 up-regulated and down-regulated lncRNAs in the differentiated stage **(E)** The ceRNA network of hub genes **(F)** A heatmap plot showing hub genes and lncRNA in differentiated stage.

To further explore the potential post-transcriptional regulation of core adipogenic genes by non-coding RNAs, we performed differential expression analysis using transcriptomic data from Day 0 and Day 7 samples. This identified 64 differentially expressed miRNAs (DE-miRNAs), including 49 upregulated and 15 downregulated, and 137 differentially expressed lncRNAs (DE-lncRNAs), comprising 77 upregulated and 60 downregulated transcripts (Fig 4A, 4B). Heatmap visualization of the top 10 up- and down-regulated candidates (Fig 4C, 4D) highlighted dynamic transcriptional remodeling of non-coding RNAs during early adipogenic transition.

To dissect the functional crosstalk between these non-coding RNAs and key adipogenic mRNAs, we integrated target prediction with expression overlap, constructing a competitive endogenous RNA (ceRNA) network composed of 7 mRNAs, 9 miRNAs, and 4 lncRNAs, with 34 predicted lncRNA–miRNA–mRNA interactions (Fig 4E). This network encompasses multiple axes centered around previously identified core genes, including LINC00847–hsa-miR-455-3p–FASN, MAPKAPK5-AS1–hsa-miR-450b-5p–FABP4, LINC00847–hsa-miR-146b-5p–ADIPOQ, ZNF667-AS1–hsa-miR-296-3p–ACSL1, LINC00324–hsa-miR-3200-5p–PLIN1, LINC00847–hsa-miR-455-3p–CIDEC, and LINC00324–hsa-miR-628-5p–LPL. These lncRNA–miRNA–mRNA axes reveal candidate post-transcriptional regulatory circuits associated with lipid accumulation and adipocyte maturation.

Temporal expression profiling between Day 0 and Day 7 further revealed coordinated patterns within the ceRNA modules (Fig 4F). For instance, the expression of FABP4 was upregulated in parallel with its corresponding lncRNA MAPKAPK5-AS1, consistent with a model in which MAPKAPK5-AS1 acts as a molecular sponge for hsa-miR-450b-5p, thereby mitigating its suppression of FABP4. Given the established roles of FABP4 in lipid handling and adipogenic programming [24], this interaction suggests a functional contribution of ceRNA-mediated derepression to the regulation of adipocyte differentiation.

### Distinct gene modules drive lipid accumulation in early adipogenesis and sustain mitochondrial function in mature adipocytes

**Fig 5:**
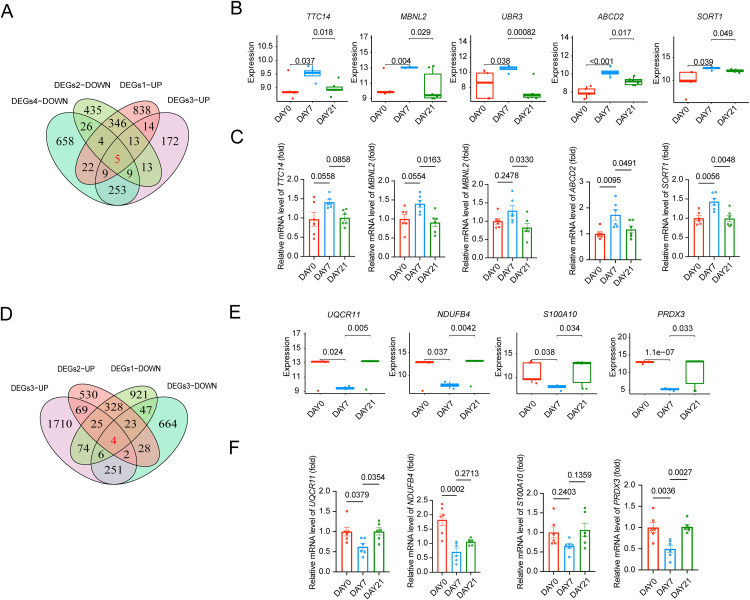
Identification of key genes for inducing adipocyte differentiation and maintaining the mature phenotype. **(A)** Venn diagram showing the key genes for inducing adipocyte differentiation **(B)** The differences of expression of key genes for inducing adipocyte differentiation among day 21, day 7, and day 0 (C) qPCR validation of TTC14, MBNL2, ABCD2, and SORT1 expression levels at Day 0, 7, and 21 during adipogenic differentiation. Data are presented as mean ± SEM (n = 6). P-values were calculated using unpaired two-tailed t-test **(D)** Venn diagram showing the key genes for maintaining the mature phenotype **(E)** The differences of expression of key genes for maintaining the mature phenotype among day 21, day 7, and day 0 (F) qPCR validation of UQCR11, NDUFB4, S100A10, and PRDX3 expression during adipogenesis (Day 0, 7, 21). P-values were calculated using unpaired two-tailed t-test. Data are shown as mean ± SEM (n = 6).

By intersecting the upregulated genes from DEGs1 and DEGs3 with the downregulated genes from DEGs2 and DEGs4, we identified five candidate regulators specifically associated with the early phase of adipocyte differentiation: TTC14, MBNL2, UBR3, ABCD2, and SORT1 (Fig 5A). These genes are involved in lipid metabolism, transcriptional control, and intracellular signaling pathways, suggesting that they may coordinate key molecular events that initiate adipogenic commitment. Temporal expression profiling revealed that these genes were prominently upregulated at Day 7 but subsequently downregulated by Day 21 (Fig 5B), a trend that was independently validated by qPCR analysis (Fig 5C). This biphasic expression pattern indicates that these genes are predominantly active during early adipogenic programming and become dispensable or repressed as cells transition toward terminal differentiation.

In contrast, four genes—UQCR11, NDUFB4, S100A10, and PRDX3—were identified by intersecting the downregulated genes in DEGs1 and DEGs3 with the upregulated genes in DEGs2 and DEGs4 (Fig 5D). These genes showed relatively low expression during early adipogenesis but were gradually upregulated as cells progressed to a mature phenotype, implying their roles in sustaining mitochondrial function and metabolic stability in differentiated adipocytes. Consistent with this, dynamic changes in their expression were observed between Day 0, Day 7, and Day 21 (Fig 5E), and were further corroborated by qPCR validation (Fig 5F). Notably, PRDX3 and UQCR11 exhibited significant and consistent differential expression across all time points (Fig 5E, 5F), reinforcing their importance in maintaining adipocyte homeostasis. The characterization of ADSCs was described as previously. Briefly, S2A Fig showed the long spindle-like adherent growth of ADSCs at low magnification. The trilineage differentiation potential of ADSCs were assessed (S2B–S2D Fig).

**Fig 6:**
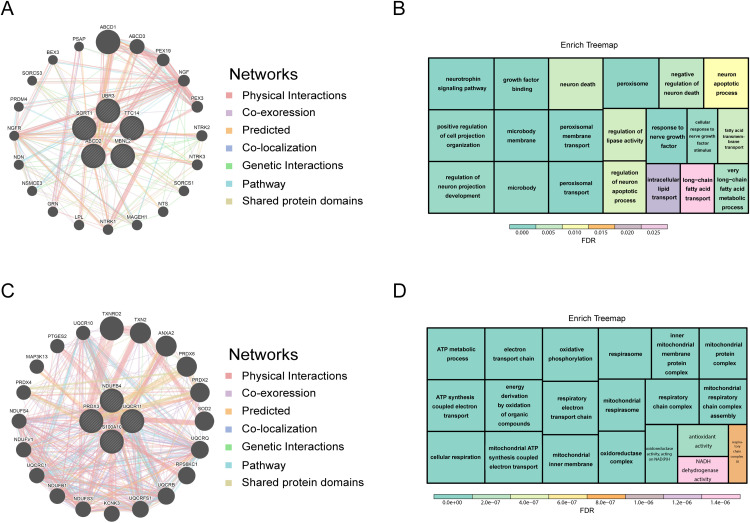
GeneMANIA analysis reveals distinct interaction networks and pathways of key genes involved in adipogenic induction and maturation. **(A)** The key genes for inducing adipocyte differentiation and their co-expression genes were analyzed via GeneMANIA **(B)** Treemap of enriched biological functions items of key genes for inducing adipocyte differentiation and their co-expression genes **(C)** The key genes for maintaining the mature phenotype and their co-expression genes were analyzed via GeneMANIA **(D)** Treemap of enriched biological functions items of key genes for maintaining the mature phenotype and their co-expression genes.

To gain insights into the potential functional interactions among these stage-specific regulators, we performed network analysis using GeneMANIA. The interaction network constructed for early-phase differentiation genes revealed a predominance of physical interactions (77.64%), followed by co-expression (8.01%), predicted interactions (5.37%), and lower proportions of co-localization, genetic interactions, pathway links, and shared domains (Fig 6A). Enrichment analysis highlighted biological processes such as very long-chain fatty acid metabolism, fatty acid transmembrane transport, and lipase activity regulation, all of which are critical to initiating lipid accumulation and remodeling during early adipogenesis (Fig 6B, S3 Table).

Similarly, the interaction network for genes associated with the maintenance of the mature adipocyte phenotype demonstrated a comparable pattern of connectivity (Fig 6C), but was instead enriched for mitochondrial functions. Specifically, pathways such as ATP synthesis coupled electron transport, mitochondrial ATP production, and the respiratory electron transport chain were significantly overrepresented (Fig 6D, S4 Table). These findings suggest that as adipocytes reach terminal differentiation, transcriptional programs shift from lipid biosynthesis toward sustaining energy metabolism and mitochondrial function, with these four genes serving as potential regulatory nodes.

## Discussion

In this study, we systematically analyzed the transcriptional dynamics of ADSCS differentiation into adipocytes by integrating bulk RNA-seq, single-cell RNA-seq, and non-coding RNA regulatory networks. Transcriptomic analysis of adipogenic differentiation in ADSCs revealed dynamic, stage-specific gene expression patterns. Early differentiation being characterised by the upregulation of the oxidative stress response (PRDX3), cytoskeletal remodelling (PALLD and WASL) and transcriptional regulation genes (FOSB), as well as the downregulation of metabolic suppressors (PCK1 and THRSP) and cytoskeletal stabilisers (RHOB) [28,29]. This suggests an early adaptive shift towards lipid accumulation and redox homeostasis. Later differentiation showed the upregulation of lipid metabolism regulators (ACSL4) and the downregulation of signalling inhibitors (SMAD3 and AKT3), indicating terminal maturation through the attenuation of TGF-β/PI3K-AKT signalling and the enhancement of oxidative stress adaptation. Single-cell RNA sequencing further delineated this progression, revealing a transitional population and distinct mature adipocyte clusters. This highlights the sequential transcriptional reprogramming that occurs from metabolic priming to terminal differentiation. These findings provide a comprehensive molecular framework for adipogenesis, identifying stage-specific regulators that could be targeted therapeutically in metabolic disorders.

Comprehensive functional enrichment analyses of adipogenesis-associated genes revealed their coordinated involvement in multiple critical biological processes for adipocyte differentiation and function. GO-BP analysis revealed their pivotal roles in fatty acid metabolism, thermoregulation and lipid localisation. Key genes such as FABP4, FABP5 and ADIPOQ were identified as critical regulators of lipid storage and energy homeostasis [30–35]. Cellular component analysis showed that they are predominantly localised to lipid droplets and organelle membranes, particularly via PLIN1 and CIDEC [36]. This suggests that they have specialised roles in lipid droplet dynamics and inter-organelle communication. Further molecular function analysis emphasised their involvement in fatty acid transport (FABP4/5), steroid metabolism (AKR1C family) and redox regulation. KEGG pathway analysis identified the peroxisome proliferator-activated receptor (PPAR) signalling pathway and lipolysis regulation as central pathways governing adipogenesis. The co-enrichment of FABP4, PLIN1 and CIDEC in lipid droplet-related pathways indicates an integrated molecular framework for regulating lipid storage, which is supported by their respective roles in fatty acid uptake (FABP4), droplet stabilisation (PLIN1) and lipid accumulation (CIDEC). Together, these findings underscore the interconnected nature of adipogenesis-associated genes in regulating lipid metabolism, energy balance, and adaptive responses. These findings shed light on the complex regulatory network of fat formation and pave the way for the development of new intervention strategies for metabolic diseases.

Our study reveals a complex ceRNA regulatory network involving seven mRNAs, nine miRNAs and four long non-coding RNAs (lncRNAs) that orchestrates adipogenic differentiation through post-transcriptional regulation. The regulatory axes identified, particularly LINC00847-hsa-miR-455-3p-FASN and MAPKAPK5-AS1-hsa-miR-450b-5p-FABP4, demonstrate how long non-coding RNAs (lncRNAs) can act as molecular sponges to regulate important adipogenic factors such as FABP4, FASN, and ADIPOQ by binding to their target microRNAs (miRNAs). Notably, MAPKAPK5-AS1-mediated regulation of FABP4 via hsa-mir-450b-5p sponging suggests a novel mechanism that controls lipid metabolism and inflammatory responses during adipocyte differentiation. Coordinated expression patterns observed between long non-coding RNAs (lncRNAs) and their target messenger RNAs (mRNAs) across differentiation stages support the functional relevance of this ceRNA network in adipogenesis [37–39]. This provides new insights into the intricate post-transcriptional regulation of adipose tissue development and potential therapeutic targets for metabolic disorders.

GeneMANIA network analysis revealed distinct functional modules for adipogenic regulation. Key gene set 1 was predominantly involved in lipid metabolic processes, including fatty acid transport and lipase regulation [40,41], while key gene set 2 was primarily associated with mitochondrial energy metabolism, such as oxidative phosphorylation and electron transport [23,42]. These findings suggest a coordinated, two-phase regulatory mechanism: the first gene cluster drives adipocyte differentiation through lipid metabolism pathways; the second maintains the function of mature adipocytes via mitochondrial bioenergetics. This highlights the stage-specific molecular networks that govern adipose tissue development and homeostasis. ADSCs can donate mitochondria to cancer cells, chiefly via tunneling nanotubes (TNTs), which enhances oxidative phosphorylation (OXPHOS), increases ATP production, and contributes to multidrug resistance (MDR) [43]. Building on our network result that key gene set 2 is enriched for mitochondrial energy metabolism, the late-stage regulators we identified—UQCR11, NDUFB4, PRDX3, and S100A10—map to pathways that could support or amplify ADSCs-to-tumor bioenergetic coupling. We therefore hypothesize that the adipogenic maturation state tunes the capacity of ADSCs to boost tumor OXPHOS through organelle exchange. This hypothesis can be tested with stage-matched co-cultures of ADSCs and cancer cells, mitochondrial tracking using donor and acceptor labels, and inhibition of TNT formation [44].

ADSCs actively shape the tumor microenvironment rather than serving as bystanders. They promote angiogenesis through a pro-angiogenic secretome (VEGF and ANGPT family signals) and can adopt perivascular traits, supporting endothelial growth and vessel stabilization [45–47]. ADSCs also exert immunomodulatory effects by releasing cytokines and extracellular vesicles that bias macrophages toward M2-like states and expand regulatory T cells, thereby dampening antitumor immunity [48–50]. In addition, ADSCs contribute to metabolic rewiring of cancer cells via lipid and adipokine supply and by organelle or metabolite exchange, including mitochondrial transfer [51]. In our study, the lipid-metabolism hub (FABP4, ADIPOQ, PLIN1, CIDEC, FASN, ACSL1, LPL, FABP5) and the late-stage regulators (UQCR11, NDUFB4, PRDX3, S100A10) align with vascular, immune, and bioenergetic pathways, suggesting that ADSCs maturation state may influence neovascularization, immune contexture, and OXPHOS-dependent phenotypes in ADSCs-rich niches.

While this study provides a systematic characterization of the transcriptional regulatory network during adipogenesis, several important directions warrant further investigation. First, expanding sample sizes and incorporating independent validation cohorts would significantly enhance the reliability and generalizability of our findings. Second, although bioinformatics analyses have identified critical regulatory genes and pathways, subsequent functional validation through gene editing (e.g., CRISPR knockout), gain-of-function (overexpression) experiments, and pathway-specific pharmacological interventions remains essential. Most importantly, integrating multi-omics approaches – including proteomics, metabolomics, and epigenomic profiling (e.g., ATAC-seq) – will facilitate the construction of a more comprehensive regulatory framework for adipocyte differentiation and potentially reveal novel regulatory molecules and their interaction mechanisms. These in-depth investigations will provide a more holistic understanding of the molecular basis of adipogenesis and may yield new therapeutic targets for metabolic disorders. In addition, although we document morphology and tri-lineage differentiation of ADSCs-like cells, a full surface-marker immunophenotype panel was not included in our study.

## Conclusions

This study provides a comprehensive transcriptional analysis of ADSC adipogenesis by integrating bulk RNA-seq and single-cell RNA-seq data. The analysis identified eight hub genes that are essential for adipogenesis, five key genes involved in inducing adipocyte differentiation, and four key genes that are crucial for maintaining the mature phenotype. Furthermore, the ceRNA network analysis unveiled potential post-transcriptional regulatory mechanisms influencing adipocyte differentiation. Additionally, the functional analysis revealed that the early-stage key genes are associated with adipogenesis-related pathways, while the late-stage key genes are linked to mitochondria-related metabolic pathways, suggesting their distinct regulatory roles in lipid metabolism and energy homeostasis. These findings offer novel insights into the molecular regulation of ADSC differentiation and provide potential targets for promoting adipogenesis in clinical applications.

## Supporting information

S1 Fig Quality control of scRNA-seq. (**A**) Violin plots showing data characterization before single-cell quality control (**B**) Violin plots showing data characterization after single-cell quality control (**C**) The top 2,000 high-variance genes (**D**) Examine and visualize PCA results with ElbowPlot.
(TIF)

S2 Fig Characterization of ADSCs. (A) The microscopic appearance of the 3rd generation of ADSCs. Scale bar 100μm. (B) ADSCs were stained with Oil red O after adipogenic differentiation. Scale bar 200μm. (C) Alizarin red staining after osteogenesis induction. Scale bar 200μm. (D) Alcian blue staining after induction. Scale bar 200μm.
(JPG)

S1 Table The list of enriched GO items of genes-related to adipocyte differentiation. (CSV)

S2 Table The list of enriched KEGG pathways of genes-related to adipogenesis. (CSV)

S3 Table The list of enriched biological functions items of key genes for inducing adipocyte differentiation and their co-expression genes. (CSV)

S4 Table The list of enriched biological functions items of key genes for maintaining the mature phenotype and their co-expression genes. (CSV)
