# Supplementary figures and images for: Integrated single-cell RNA-seq and bulk RNA-seq analysis to investigate key adipogenesis genes in adipose-derived stem cells
Source: PLoS One. 2025 Dec 1;20(12):e0335152. doi: 10.1371/journal.pone.0335152 (PMC12668495; doi:10.1371/journal.pone.0335152)

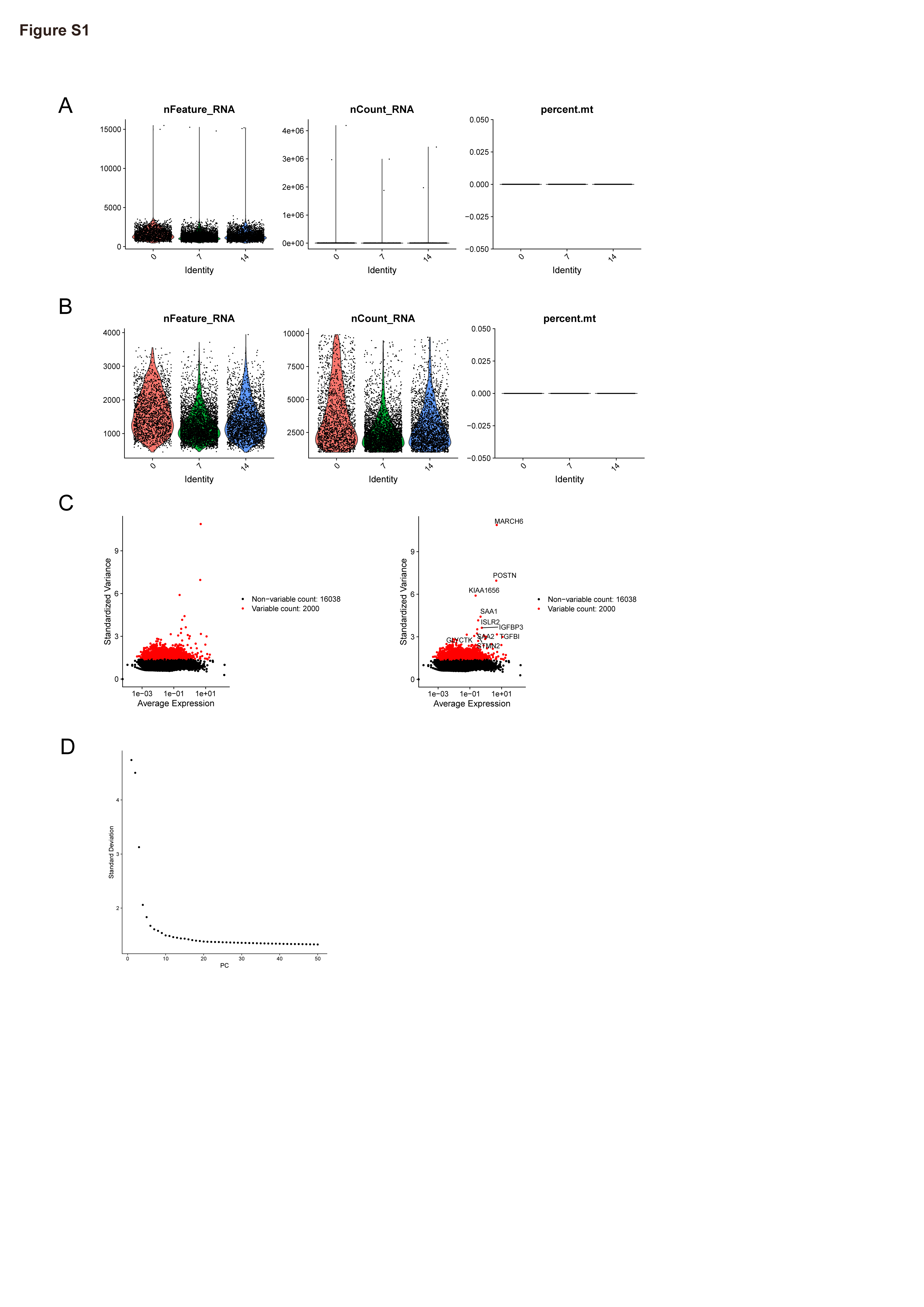

Supplement: S1 Fig — (A) Violin plots showing data characterization before single-cell quality control (B) Violin plots showing data characterization after single-cell quality control (C) The top 2,000 high-variance genes (D) Examine and visualize PCA results with ElbowPlot. (TIF) [file pone.0335152.s001.tif]

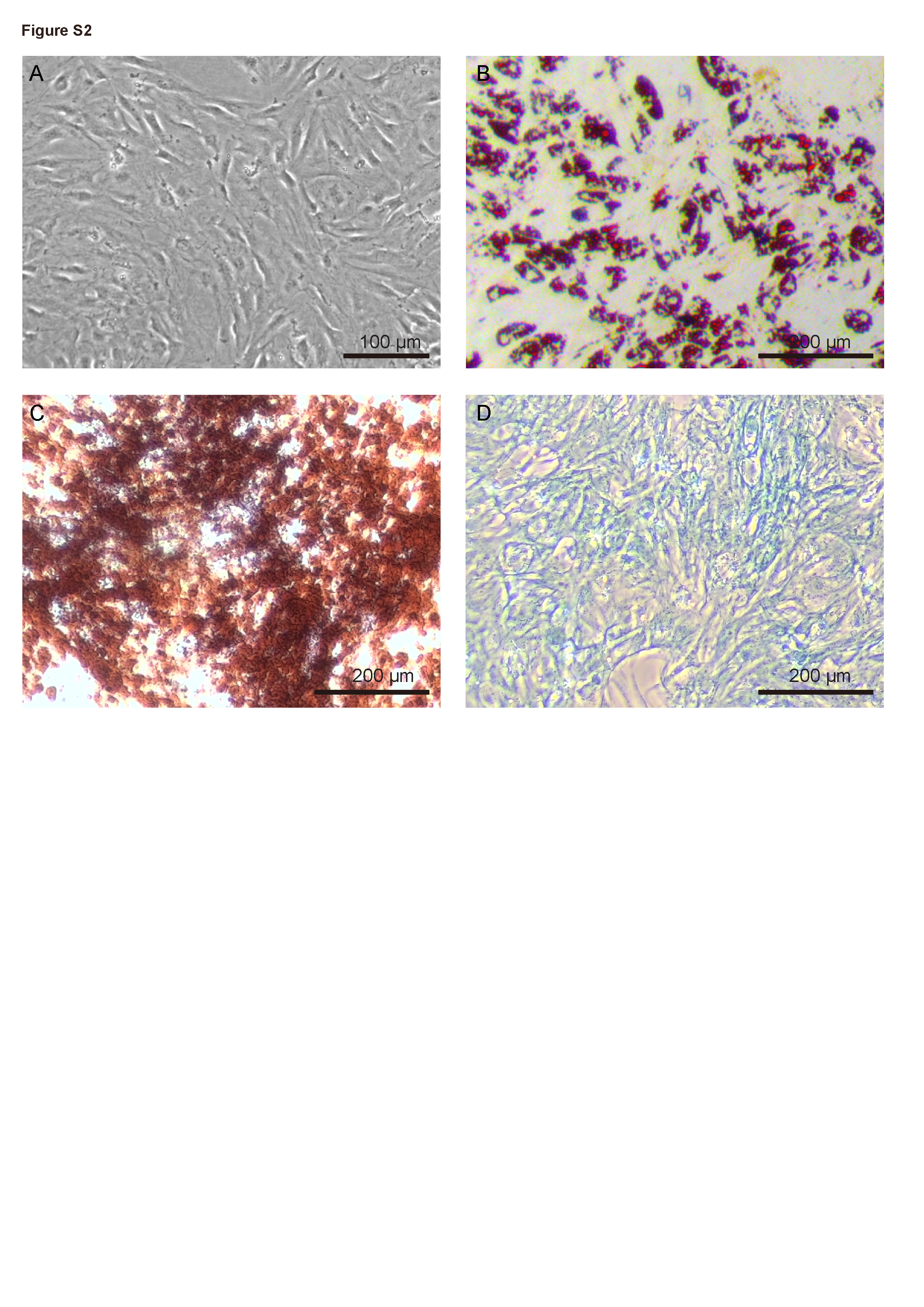

Supplement: S2 Fig — (A) The microscopic appearance of the 3rd generation of ADSCs. Scale bar 100μm. (B) ADSCs were stained with Oil red O after adipogenic differentiation. Scale bar 200μm. (C) Alizarin red staining after osteogenesis induction. Scale bar 200μm. (D) Alcian blue staining after induction. Scale bar 200μm. (JPG) [file pone.0335152.s002.jpg]
